# Supplementary material for: Exploring the Antimicrobial Resistance Profile of Salmonella typhi and Its Clinical Burden
Source: Antibiotics (Basel). 2024 Aug 14;13(8):765. doi: 10.3390/antibiotics13080765 (PMC11350866; doi:10.3390/antibiotics13080765)
Supplement: Supplementary file 1 [file antibiotics-13-00765-s001.zip › antibiotics-3096155-supplementary.pdf]

Supplementary Figure S1. The WHO-based proforma used for the collection of blood samples

MR #: ____________________ EPID #: ____________________

Sentinel site information | **Province:**

Name of Health facility:

District: | **Date of investigation:**

Patient's information | 1. **Name:**

1. **Father / Husband Name:** | 2. **Sex:** *Male* *Female*

3. **Age**_________ If less than 2 Yrs.: _____ Months **Mobile Phone Number:**

4. **Address:** *Village/Mohallah:* ____________________ */UC#:* ____________________

Town/Tehsil: ____________________ *District:* ____________________.

Type of Locality: Urban Peri Urban Rural

Drinking Water Source: Municipality Ground water (Well), Surface water (Shallow well, streams etc.) Other

If other, please specify: ____________

5. **History of Fever for at least 03 days during last week present?** Yes No

If No, Stop investigation and exclude the case.

6. **Has the patient received Typhoid conjugate vaccine (TCV):** Yes: No Unknown

If Yes, Number of Doses: Date of Last Vaccination: ____/_____/________

History of current illness:

1 **Date of Onset of initial illness symptoms:**
____/____/____

1. **Symptoms:**

History of Fever ☐ Yes ☐ No ☐ Unknown

(related to current visit)

4. **Medication prior to current medical evaluation** *(check all that apply)*

Medicine	Yes	No	Unknown
Antibiotics			

If Yes

Name of Antibiotics:

2. **Household contact with a confirmed case of typhoid or paratyphoid fever in 28 days before onset of illness:**
Yes *No*

If Yes, please mention details:

3. **Travel history within last 28 days before onset of illness for persons to endemic settings:** *Yes* *No*

If yes, please mention details of travelled destination:

Hospitalization:

Was patient admitted to Hospital during current illness?

☐ *Yes* ☐ *No*

Date of admission: ____/_____/________

Outcome of Case: Cured Complicated Died

Were there any associated complications?

☐ *Yes* ☐ *No*

If yes, please mention details:

Name & Designation of the investigating Officer:

Laboratory Part: *(To be filled by Lab)*

1. **Was blood sample taken?** *Yes* *No*

2. **Date of Sample collection:** ____/_____/__________

3. **Date of Sample sent to the Lab:** ____/_____/__________

4. **Date of Sample received in Lab:** _____/_____/________

5. **Date of Result:** _____/_____/_____

Blood Culture Result:

Positive Negative Not Done

If Positive, Type of isolate: Salmonella Typhi Salmonella Para typhi (A, B, C), Other:

Was antibiotic Sensitivity Testing Performed?

Yes

No

If Yes, Was the Organism Resistant to: (use ✓)

Antibiotic	Yes	No	Not Tested
Chloramphenicol			
Ampicillin			
Co-Trimoxazole			
Fluoroquinolones			
Cefixime/Ceftriaxone			
Azithromycin			
Meropenem			

Final Classification

Discarded Salmonella Positive

If Salmonella Positive, Specify Type:

XDR MDR Non-resistant inconclusive

Name & Designation of Lab focal person: **Date:**

Supplementary Table S1. WHO Classification of Typhoid fever cases by drug resistance status.

**WHO classification for sensitive and resistant *S. Typhi***	
**Non-resistant *S. typhi***	If the *S. typhi* isolates are sensitive to first-line and third-line antibiotics (cefixime and ceftriaxone) and with or without susceptibility to second-line antibiotics
**Multi-drug Resistant (MDR) *S. typhi***	If the *S. typhi* isolates are resistant to first-line antibiotics but sensitive to third-line antibiotics and with or without resistance to second-line antibiotics
**Extensively drug-resistant (XDR) *S. typhi***	Resistance to all three lines of drugs but still sensitive to a few antibiotics like carbapenems and Azithromycin
**Pan drug-resistant (PDR) *S. typhi***	Resistant to all antibiotics or any clinically available antibiotics called PDR
